# Supplementary material for: Assessment of the Biocontrol Potential of Bacillus velezensis WL–23 against Kiwifruit Canker Caused by Pseudomonas syringae pv. actinidiae
Source: Int J Mol Sci. 2023 Jul 16;24(14):11541. doi: 10.3390/ijms241411541 (PMC10380555; doi:10.3390/ijms241411541)
Supplement: Supplementary file 1 [file ijms-24-11541-s001.zip › ijms-2490852-supplementary.pdf]

Supplementary Material

Assessment of the biocontrol potential of *Bacillus velezensis* WL-23 against kiwifruit canker caused by *Pseudomonas syringae* pv. *Actinidiae*

Bingce Wang 1, Yushan Guo 1, Xuetang Chen 1, Jilin Ma 1, Xia Lei 1, Weizhen Wang 1 and Youhua Long 1,2,*

[1]College of Agriculture, Research Center for Engineering Technology of Kiwifruit, Institute of Crop Protection, Guizhou University, Guiyang 550025, China.

[2]Teaching Experimental Field of Guizhou University, Guiyang 550025, China.

*** Correspondence:** Youhua Long
gzlyh126@126.com

Supplementary Table S1. Reference isolates used in the present study and their GenBank accession numbers.

Species	Culture Accession	GenBank Accession	
		16S rDNA	*gyrA*
Bacillus amyloliquefaciens	DSM7 [T]	FN597644	FN597644
Bacillus atrophaeus subsp. *globigii*	BSS	CP007640	CP007640
Bacillus glycinifermentans	GO-13 [T]	LECW00000000	LECW00000000
Bacillus halotolerans	ATCC 25096 [T]	LPVF00000000	LPVF00000000
Bacillus haynesii	NRRL B-41327 [T]	NR-157609	AZYP00000000
Bacillus haynesii	P19	CP059494	CP059494
Bacillus inaquosorum	KCTC 13429 [T]	AMXN00000000	AMXN00000000
Bacillus licheniformis	DSM 13 [T]	LR594217	AE017333

Bacillus mojavensis	RO-H-1 [T]	AFSI00000000	AFSI00000000
Bacillus nakamurai	NRRL B-41091 [T]	KU836854	LSAZ00000000
Bacillus siamensis	KCTC 13613 [T]	OM995809	AJVF00000000
Bacillus sonorensis	NBRC 101234 [T]	BCVZ00000000	BCVZ00000000
Bacillus subtilis	NCIB 3610 [T]	MT421926	CP020102
Bacillus subtilis subsp. *spizizenii*	NRRL B-23049 [T]	NR-024931	EU138602
Bacillus subtilis subsp. *spizizenii*	TU-B-10 [T]	NC-016047	NC-016047
Bacillus swezeyi	NRRL B-41294 [T]	NR-157608	MRBK00000000
Bacillus thuringiensis serovar berliner	ATCC 10792 [T]	CM000753	CM000753
Bacillus vallismortis	DSM 11031 [T]	NR-024696	CP026362
Bacillus vanillea	XY18 [T]	KF986320	LAGT00000000
Bacillus velezensis	WLYS23	CP055160	CP055160

Note: The order of the strains was alphabetical. T = type strain.
